# Human Breast Cancer Tissues Contain Abundant Phosphatidylcholine(36∶1) with High Stearoyl-CoA Desaturase-1 Expression

**DOI:** 10.1371/journal.pone.0061204

**Published:** 2013-04-16

**Authors:** Yoshimi Ide, Michihiko Waki, Takahiro Hayasaka, Tomohisa Nishio, Yoshifumi Morita, Hiroki Tanaka, Takeshi Sasaki, Kei Koizumi, Ryoichi Matsunuma, Yuko Hosokawa, Hiroyuki Ogura, Norihiko Shiiya, Mitsutoshi Setou

**Affiliations:** 1 Department of Surgery I, Hamamatsu University School of Medicine, Hamamatsu, Japan; 2 Department of Cell Biology and Anatomy, Hamamatsu University School of Medicine, Hamamatsu, Japan; 3 Department of Laboratory Medicine, Hamamatsu University School of Medicine, Hamamatsu, Japan; 4 Department of Anatomy and Neuroscience, Hamamatsu University School of Medicine, Hamamatsu, Japan; Centro Nacional de Investigaciones Oncológicas (CNIO), Spain

## Abstract

Breast cancer is the leading cause of cancer and mortality in women worldwide. Recent studies have argued that there is a close relationship between lipid synthesis and cancer progression because some enzymes related to lipid synthesis are overexpressed in breast cancer tissues. However, lipid distribution in breast cancer tissues has not been investigated. We aimed to visualize phosphatidylcholines (PCs) and lysoPCs (LPCs) in human breast cancer tissues by performing matrix assisted laser desorption/ionization-imaging mass spectrometry (MALDI-IMS), which is a novel technique that enables the visualization of molecules comprehensively. Twenty-nine breast tissue samples were obtained during surgery and subjected to MALDI-IMS analysis. We evaluated the heterogeneity of the distribution of PCs and LPCs on the tissues. Three species [PC(32∶1), PC(34∶1), and PC(36∶1)] of PCs with 1 mono-unsaturated fatty acid chain and 1 saturated fatty acid chain (MUFA-PCs) and one [PC(34∶0)] of PCs with 2 saturated fatty acid chains (SFA-PC) were relatively localized in cancerous areas rather than the rest of the sections (named reference area). In addition, the LPCs did not show any biased distribution. The relative amounts of PC(36∶1) compared to PC(36∶0) and that of PC(36∶1) to LPC(18∶0) were significantly higher in the cancerous areas. The protein expression of stearoyl-CoA desaturase-1 (SCD1), which is a synthetic enzyme of MUFA, showed accumulation in the cancerous areas as observed by the results of immunohistochemical staining. The ratios were further analyzed considering the differences in expressions of the estrogen receptor (ER), human epidermal growth factor receptor 2 (HER2), and Ki67. The ratios of the signal intensity of PC(36∶1) to that of PC(36∶0) was higher in the lesions with positive ER expression. The contribution of SCD1 and other enzymes to the formation of the observed phospholipid composition is discussed.

## Introduction

Breast cancer is the leading cause of cancer and cancer related mortality in women worldwide [1]. Recently, the activation of lipid metabolism in breast cancer cells has been increasingly recognized as a hallmark of carcinogenesis [2], [3]. In particular, phosphatidylcholines (PCs) are generally the most abundant phospholipid species in mammalian cells, and PC synthesis and metabolism in cancer progression have been investigated [4]. Aberrancy in PC metabolism, which is mainly through the increased degradation of PCs, was indicated in a study using nuclear magnetic resonance for the analysis of breast cancer cell lines; however, they did not distinguish the acyl chain structures of the PCs [5]–[7]. The characterization of breast cancer tissues from patients by differentiating among molecular species of PCs has been reported by using gas chromatography [8] and liquid chromatography/mass spectrometry [9]. Biomarker investigation by lipidomic analysis including several PC species has been proposed for several PC species as putative diagnostic markers and therapeutic targets [9].

In this report, we apply matrix assisted laser desorption/ionization (MALDI)-imaging mass spectrometry (IMS), which is a recently developed analysis methodology [10], to analyze breast cancer tissues. MALDI-IMS enables biomolecules on tissue samples to be ionized while preserving their positional information by 2-dimensional laser scanning. The ionized biomolecules can be simultaneously analyzed by using a time-of-flight type mass spectrometer and identified according to their mass-to-charge ratio (*m/z*). The distribution of a target biomolecule is 2-dimensionally visualized as the relative ratio of the signal intensity among the measurement points of the tissue section [11]–[13].

**Figure 1:**
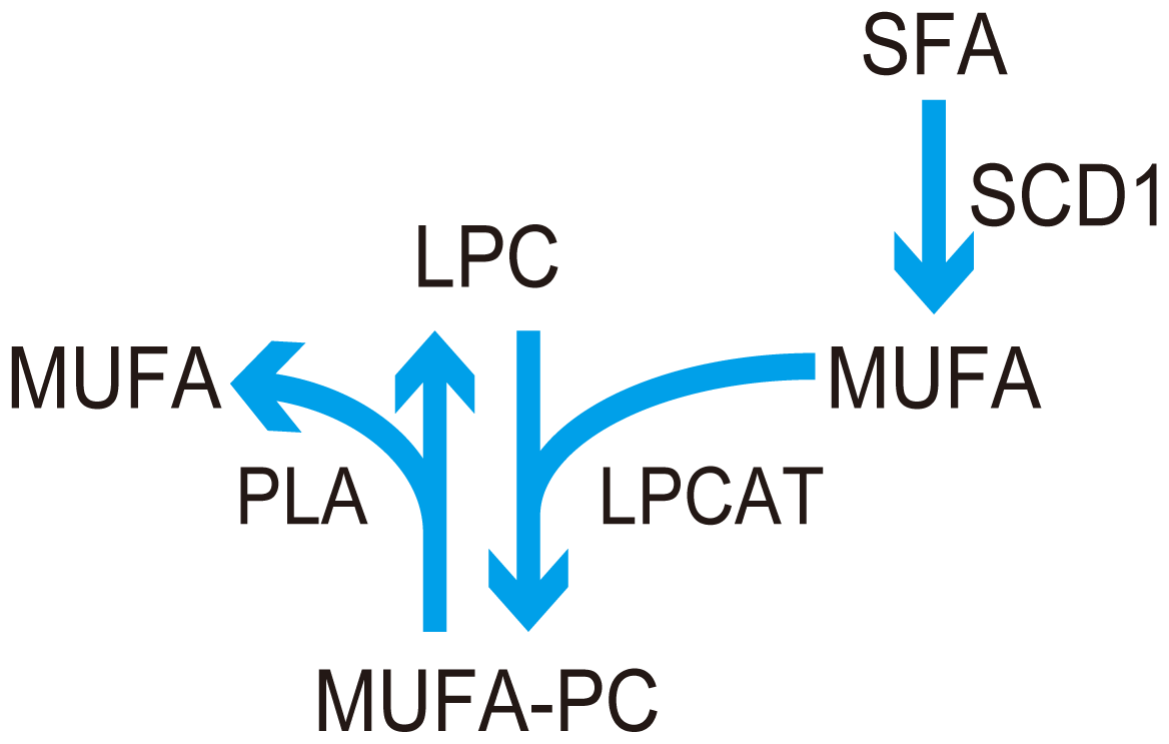
Pathway for synthesizing MUFA-PCs catalyzed by LPCAT and LPC. Endogenously synthesized mono-unsaturated fatty acids (MUFAs) are converted from saturated fatty acids (SFA) by the catalyzing effect of stearoyl-CoA desaturase-1 (SCD1). A MUFA is added to a lyso-phosphatidylcholine (LPC) through lysophosphatidylcholine acyltransferase (LPCAT) to produce a phosphatidylcholine (PC) that contains MUFA (MUFA-PC). A MUFA-PC is degraded to a LPC and a MUFA by phospholipase A1 (PLA1) and PLA2.

Herein, we mainly visualize some specific PCs composed of mono-unsaturated fatty acids (MUFAs) and saturated fatty acids (SFAs), since the incorporation of these fatty acid into PCs and the proportion of the PCs as end products in cancer cells are not well understood. Stearoyl-CoA desaturase-1 (SCD1) is a microsomal enzyme that regulates the conversion of SFA (palmitic [16∶0] and stearic acid [18∶0]) into MUFA (palmitoleic [16∶1] and oleic acid [18∶1], respectively) [14]–[16] (Figure 1) and is suggested to play an important role in cancer progression [17]. Although a recent study has proposed that higher SCD1 expression is a poor prognostic marker for breast cancer [18], reports on the use of immunohistochemical analysis using human breast cancer tissues are limited [19]. Therefore, we examine SCD1 expression and its correspondence with the composition of phospholipids as the final products of sequential enzymatic reactions, including desaturation.

In this report, we attempt to visualize phospholipids in breast cancer tissues, and describe the distributions by means of signal intensities of the molecules and the ratios by discriminating the area containing cancer cells and the rest of the sections. We evaluate the expression of SCD1 proteinin cancer cells to consider the relationship between desaturation and lipid composition. Lipid composition is further analyzed with regard to estrogen receptor (ER), human epidermal growth factor receptor 2 (HER2), and Ki67 expression.

## Methods

### Ethics Statement

All the experiments in this study were specifically approved by the Ethics Committee at the Hamamatsu University School of Medicine. Informed consent was obtained in written form from each patient before performing each operation. One patient under 20 years of age was involved in our study and for her informed consent was obtained in written form from her parents as well. The subjects consented to cooperate after they were informed that they would not incur any disadvantage, that they could resign from the study, that the researchers were obliged to protect their privileged information, and that their identities would not be revealed.

### Subjects

Tissue samples were obtained from 29 patients (sample No. 1–29; n = 29), who underwent surgery at our hospital (University Hospital, Hamamatsu University School of Medicine). Only 1 sample (sample No. 20) was from a breast fibroadenoma that had been resected from a patient who did not have cancer, while the other 28 samples (No. 1 to 29 except 20) were resected from patients who had been diagnosed with breast cancer on the basis of pathological examinations by using formalin fixed and paraffin embedded (FFPE) tissue sections. All of the samples in this study were reexamined microscopically by 2 authors (YI and HO). Since 1 out of 28 samples that were obtained from cancer patients did not contain any cancer cells (sample No. 28), 27 samples were used for the analysis of cancer lesions. All of the patients were Japanese women who were aged 16–87 y (mean age, 59.3 y). One out of the 28 breast cancer patients received preoperative systemic therapy (sample No. 10). Three out of the 28 patients had non-invasive ductal carcinomas (sample No. 3, 18, and 24) and the others had invasive carcinomas.

Information on the expression of ER, HER2, and Ki67 was also obtained from the pathological records of clinical specimen in the patients’ medical records. Specimens showing positive staining for ER in >10% of the total cancer cells were defined as being positive ER lesions (sample No. 2, 3, 6–9, 11, 13–18, 22–27, and 29; n = 20). The others were classified into negative ER lesions (sample No. 1, 4, 5, 10, 12, 19, and 21; n = 7). HER2 positivity was determined according to the guidelines of the American Society of Clinical Oncology and the College of American pathologists [20]. Tumor cells with cell membranes that were completely stained by HER2 antibody comprising of >30% of the total cancer cells were given an HER2 scoring of 3+ and recognized as being HER2 positive lesions. A fluorescence in situ hybridization test was performed for the lesions with HER2 scores of 2+ and lesions with a signal ratio of over 2.2 were recognized as being HER2 positive (sample No. 1, 8, 11, 12, 19, and 22; n = 6). The other lesions were considered as being HER2 negative (sample No.2–7, 9, 10, 13–18, 21, 23–27, and 29; n = 21). Seventeen of the patients were ER and/or progesterone receptor (PgR) positive and HER2 negative (sample No. 2, 3, 6–9, 11, 13–18, 22–27, and 29; n = 17), 3 were ER and/or PgR positive and HER2 positive (sample No. 8, 11, and 22; n = 3), 3 were both ER and PgR negative and HER2 positive (sample No. 1, 12, and 19; n = 3), and 4 were triple negative (sample No. 4, 5, 10, and 21; n = 4). Lesions with a Ki67 index of over 20% were recognized as belonging to the higher Ki67 group and fewer than 20% belonged to the lower group [21]. Information on the Ki67 labeling index of 21 out of the 27 lesions was obtained from medical records; 14 lesions (sample No. 2, 3, 6, 7, 9, 10, 13, 16, 18, 24–27, and 29; n = 14) were classified as belonging to the lower Ki67, and 7 belonged to the higher Ki67 (sample No. 4, 5, 8, 11, 12, 15, and 17; n = 7).

### Chemicals

Calibration standard peptides (Bradykinin fragment 1–7 and Angiotensin II) and 2, 5-dihydroxybenzoic acid (DHB) were purchased from Sigma-Aldrich (St. Louis, MO, USA).

### Sample Preparation

Samples for analysis by IMS were immediately frozen in liquid hexanes to minimize degradation and were kept at −80°C. The specimens were sliced into 10-µm thick sections using a cryostat (CM1950; Leica, Wetzler, Germany). During sectioning, the temperature in the cryostat was maintained at −20°C. Then, the slices that were to be used for MALDI-IMS were mounted onto indium-tin-oxide (ITO)-coated glass slides (Bruker Daltonics, Charlotte, NC, USA), and the adjacent section was used for counter staining with hematoxylin and eosin (HE). The samples that were used for IMS were also stained with HE after analysis. Two authors (YI and HO) performed a microscopic examination of the HE stained glass slides of adjacent sections and those after IMS analysis. Breast cancer cells are identified from the findings that its cytoplasm is often abundant and eosinophilic and its nuclei may be regular, uniform or highly pleomorphic with prominent, often multiple, nucleoli, mitotic activity may be virtually absent or extensive [22]. Regions that were regarded as exhibiting insufficient pathological findings, whether benign or malignant, were excluded from the analysis.

### IMS Analysis

A thin matrix layer was applied to the surface of the samples that were placed on ITO-coated glass slides by using a vapor deposition device (RK27-4069; Shimadzu Corporation, Kyoto, Japan) as previously reported [23].

MALDI-IMS was performed by using a MALDI-TOF/TOF-type instrument (Ultraflex II TOF/TOF; Bruker Daltonics). The machine was equipped with a 355-nm Nd: YAG laser that operated at a repetition rate of 200 Hz and was controlled by flexControl 2.4 software (Bruker Daltonics). The data were acquired in the range of *m/z* 400 (500 in 6 samples)-1000 by using step sizes of 90–130 µm for the samples in the positive ion mode. All of the spectra were acquired automatically using FlexImaging 2.1 software (Bruker Daltonics). The mass spectra were calibrated externally by using the bradykinin fragment 1–7 ([M+H]^+^, *m/z* 757.39916), angiotensin II ([M+H]^+^, *m/z* 1046.54180), and DHB ([M+H]^+^, *m/z* 155.03000). Imaging reconstruction was performed using the FlexImaging 2.1 software (Bruker Daltonics).

### Lipid Analysis

Twenty-nine specimens from 29 patients were provided for IMS analysis. After measurement and data reconstruction, we set regions of interest (ROIs) of approximately 500 µm×500 µm to obtain mean of signal intensities at the specified regions. We defined ‘cancerous areas’ as areas that contain cancer cells and cancer-free ‘reference areas’ as the rest of the measured areas on the sections, referring the HE staining of the section.

For the data analysis presented in Figures 3 and 5, we set 27 ROIs in the cancerous areas and 8 ROIs in the reference areas. All ROIs in cancerous and reference areas were carefully set by following the microscopic reexamination that was mentioned in the part of Sample preparation. Twenty-one cancerous ROIs were set on 21 sections, as each section contained 1 cancerous area (sample No. 1–9, 11, 13, 15–19, 21, 24, 25, 27, and 29). Two reference ROIs were set on 2 sections (sample No. 20 and 28). For the remaining 6 sections, we set both 1 cancerous ROI and 1 reference ROI on each section (sample No. 10, 12, 14, 22, 23, and 26).

For the data analysis for Figures S2 and S4, we used the data obtained from the measurement of 6 sections (sample No. 10, 12, 14, 22, 23, and 26). Three cancerous ROIs and 3 reference ROIs were set in each sample.

The signal intensity of each extracted *m/z* was calculated and exported by using FlexImaging 2.1 software. Previous reports [24], [25] and a mass library (http://www.lipidmaps.org/data/structure/LMSDSearch.php) were used as references to make assignments for the molecular ions.

Most fatty acids that are produced in mammals usually have 16–18 carbons [26]. Based on this fact, the PCs whose number of carbons were 32, 34, or 36 and whose degree of unsaturation was 0 or 1 were defined as being representative PCs. Lysophosphatidylcholines (LPCs) that could be substrates for the generation of these PCs were considered to be representative LPCs.

The intensities of representative PCs and LPCs were recorded and compared between cancerous areas and reference areas. All of the statistical analyses were performed using Statcel2 software (OMS Ltd., Saitama, Japan). Welch’s *t*-test (p value of <0.005) was used to perform statistical analysis on the peak intensity values of the cancerous and reference areas.

The ratios of MUFA-PCs to SFA-PCs and LPCs were compared between the cancerous areas and the reference areas. The ratio was calculated from the recorded intensities and analyzed by employing Welch’s *t*-test (p value of <0.005) and using Statcel2 software.

A paired *t*-test (p value of <0.01) was used to perform statistical analysis of the intensities between the cancerous areas and reference areas in the supplemental analysis by considering the two areas on a single section as being a pair. Since the substantial sample size (the number of specimens provided to the analysis) in this comparison was small as six in each group, we used p value of 0.01 for threshold for examination of significance only in this analysis.

To investigate the relationship between clinical marker expression and the ratios of MUFA-PCs to SFA-PCs and LPCs in the cancerous areas, the ratio of MUFA-PCs to SFA-PCs and LPCs found by the difference of expression of ER, HER2, and Ki67 were calculated. Statistical analyses were performed by using Welch’s t-test (p value of <0.005).

### Immunohistochemical Staining

Immunohistochemical staining was performed to detect SCD1 expression. The formalin-fixed and paraffin-embedded specimens were sliced into 4–5 µm thick slices and mounted onto slide glass. The sections were de-paraffinized in xylene and rehydrated in a graded ethanol series. For antigen retrieval, the sections were heated in Tris-ethylenediaminetetraacetic acid buffer (pH 9.0) for 40 min. The sections were immersed in 3% H_2_O_2_/methanol for 5 min to quench endogenous peroxidase. Next, they were pre-incubated with 3% normal serum and then incubated with mouse monoclonal antibody against SCD1 (1∶50; GeneTex, Irvine, CA, USA) overnight at 4°C. Following this, the sections were allowed to react with horseradish peroxidase-conjugated goat secondary antibody against mouse IgG (1∶200, Vector Laboratories, Burlingame, CA, USA) for 2 h at 4°C. The sections were visualized by using a DAB substrate kit (Vector Laboratories), according to the manufacturer’s instructions. Basal epithelial cells in the mammary gland were used as an inner negative control for SCD1 staining, by referring the previous report on SCD1 staining for prostate cancer tissues [27]. Apocrine cells in the skin were used as an inner positive control. Samples that were regarded as exhibiting insufficient staining or non-specific staining were excluded from the analysis. Three cancerous areas (sample No.16–18) were excluded for the analysis because of insufficient staining or non-specific staining. The remaining 24 cancerous areas and 8 reference areas were judged as areas that were stained appropriately. The expression levels for the positive cells were assessed by using Image J (NIH, Bethesda, MD, USA).

For the comprehensive analysis shown in Figure 4c, the expression levels of SCD1 were compared between 24 cancerous areas (sample No. 1–15, 19, 21–27, and 29; n = 24) and 8 reference areas (sample No. 10, 12, 14, 20, 22, 23, 26, and 28; n = 8). For sub-analysis in consideration of the ER status that is shown in Figure 6, cancerous lesions with positive ER (sample No. 2, 3, 6–9, 11, 13–15, 22–27, and 29; n = 17) and those with negative ER (sample No. 1, 4, 5, 10, 12, 19, and 21; n = 7) were analyzed. For sub-analysis considering HER2 status, cancerous lesions with negative HER2 (sample No. 2–7, 9, 10, 13–15, 21, 23–27, and 29; n = 18) and those with positive HER2 (sample No. 2, 8, 11, 12, 19, and 22; n = 8) were used. For sub-analysis considering Ki67, cancerous lesions with lower Ki67 (sample No. 2, 3, 6, 7, 9, 10, 13, 24, 25, 26, 27, and 29; n = 12) and those with higher Ki67 (sample No. 4, 5, 8, 11, 12, and 15; n = 6) were used. Welch’s *t*-test (p value of <0.005) was used for the statistical analysis.

To examine the presence of bias in the distribution of SCD1 staining intensity and the MUFA-PC to SFA-PC ratio, the values from all samples that were correctly stained with SCD1 antibody were plotted two-dimensionally. A borderline was set to divide the plots and frequencies into quadrants that were subjected to Pearson’s chi-square test (p value of <0.005) to examine if their distributions were different from the theoretical one. The presence of such borderlines on PCs with 32, 34, and 36 acyl carbons was examined by conducting the above procedure.

## Results

### Visualization by MALDI-IMS of PCs in Human Breast Cancer Tissues

**Figure 2:**
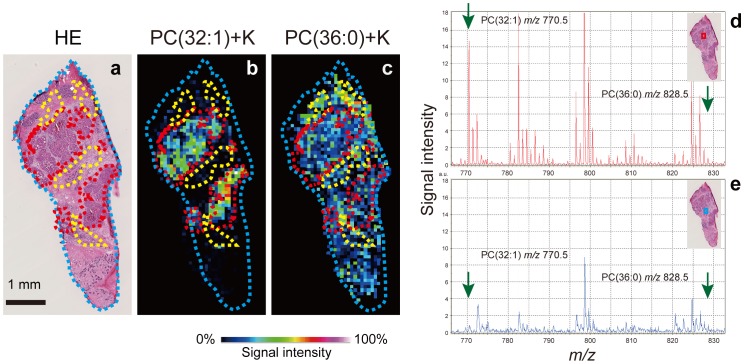
Different species of phosphatidylcholine were visualized on a breast cancer tissue specimen. (a) The areas that are circled with broken red lines in the adjacent hematoxylin and eosin (HE) stained images show cancerous areas and those that are circled with broken yellow lines show stromal tissue around the ducts. (b) A distribution image of PC(32∶1)+K by MALDI-IMS shows the accumulation of PC(32∶1)+K in the cancerous areas. (c) A distribution image of PC(36∶0)+K shows the accumulation of PC(36∶0)+K in the stromal tissue around the ducts. (d and e) Mass spectra obtained for a cancerous area shows different patterns from those of a reference area. The red and blue squares in the HE stained image in the inset shows the analytical points in a cancerous area and a reference area, respectively.

We succeeded in visualizing some molecules by IMS. Figure 2 shows the images that were obtained on performing MALDI-IMS and an HE staining of the adjacent section. Since the samples that were used for IMS analysis were severely destroyed by laser irradiation, HE-stained images of adjacent sections were used for the identification of cancerous areas and are presented in the figures. The samples after IMS analysis were also stained by HE and ascertained by microscopic examination to closely resemble the adjacent tissue sections (Figure S1). The cancerous areas are indicated by broken red lines, and the stromal tissues that were around the ducts are circled with broken yellow lines in the HE-stained image (Figure 2a). Figure 2b and 2c depict typical images that were obtained by MALDI-IMS, which were used to visualize the maldistribution of PC(32∶1)+K (Figure 2b) and PC(36∶0)+K (Figure 2c) corresponding predominantly to the cancerous areas and the stromal tissues around the ducts, respectively. Figures 2d and 2e show an example of mass spectra obtained from the cancerous area (Figure 2d) and the stromal region (Figure 2e) of the section. The analyzed areas are indicated by blue and red squares on the HE-stained image of the adjacent section. The mass spectra of this sample showed that the signal of PC(32∶1)+K at *m/z* 770.5 was predominantly detected in the cancerous areas, while the ion signal was substantially reduced in the stromal region. On the other hand, the signal of PC(36∶0)+K at *m/z* 828.5 did not show higher accumulation in the cancerous area.

### The Ratios of MUFA-PCs Compared to SFA-PCs were Significantly Higher in the Cancerous Areas

We asked whether there might be any molecules characteristically observed in the cancer cell regions. We defined reference areas as regions excluding cancerous areas that were identified pathologically. Then, the intensities of each molecule in the cancerous areas were compared to those in the reference areas. Differences in the ratios of MUFA-PCs to SFA-PCs were examined between the cancerous and reference areas as well.

**Figure 3:**
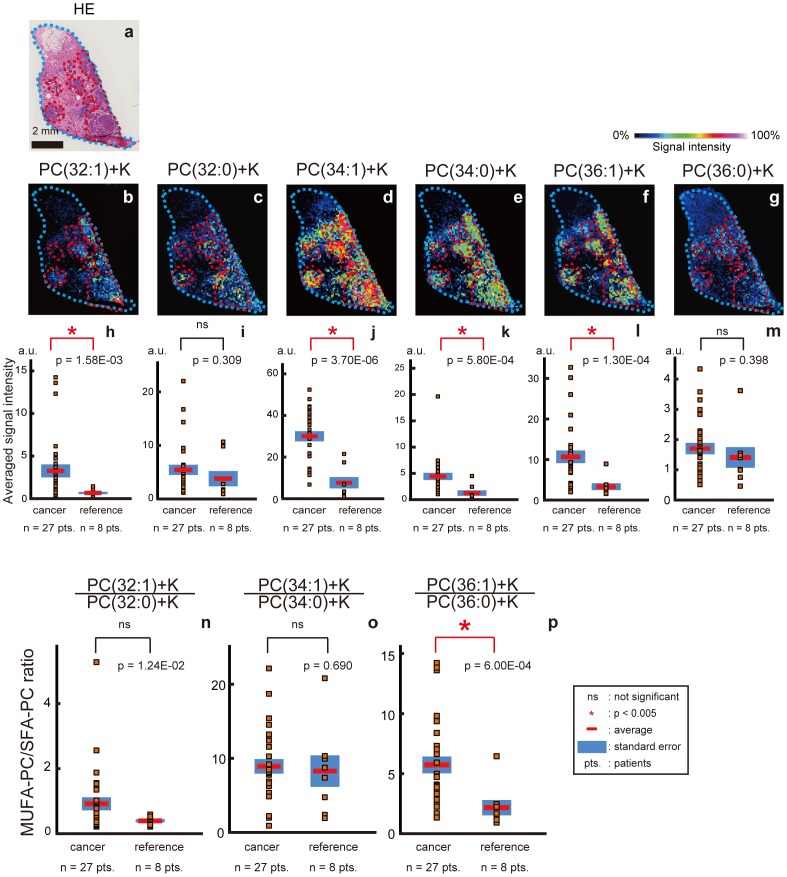
The amount of MUFA-PCs relative to SFA-PCs was significantly higher in cancerous areas. (a) The areas that are circled with broken red lines in the hematoxylin and eosin stained image show the cancerous areas. (b) A distribution image of PC(32∶1)+K. (C) A distribution image of PC(32∶0)+K. (d) A distribution image of PC(34∶1)+K. (e) A distribution image of PC(34∶0)+K. (f) A distribution image of PC(36∶1)+K. (g) A distribution image of PC(36∶0)+K. (h) Plot of the intensities of PC(32∶1)+K. (i) Plot of the intensities of PC(32∶0)+K. (j) Plot of the intensities of PC(34∶1)+K. (k) Plot of the intensities of PC(34∶0)+K. (l) Plot of the intensities of PC(36∶1)+K. (m) Plot of the intensities of PC(36∶0)+K. (n) Plot of the ratios of PC(32∶1)+K to PC(32∶0)+K. (o) Plot of the ratios of PC(34∶1)+K to PC(34∶0)+K. (p) Plot of the ratios of PC(36∶1)+K to PC(36∶0)+K.

The areas that are circled with broken red lines on the HE staining image show the cancerous areas (Figure 3a). The heat map images of each molecular ion (Figures 3b to 3g) and a comparison of the signal intensities of the cancerous areas and the reference areas by signal intensity plots (Figures 3h, j, k, and l) also showed high degrees of accumulation of PC(32∶1)+K (Figures 3b and h; p = 1.58E-03), PC(34∶1)+K (Figure 3d and j; p = 3.70E-06), PC(34∶0)+K (Figures 3e and k; p = 5.80E-04), and PC(36∶1)+K (Figures 3f and l; p = 1.30E-04) in the cancerous areas. The ratio of PC(36∶1)+K to PC(36∶0)+K (Figure 3p) was significantly higher in the cancerous areas than in the reference areas (p = 6.00E-04), while the ratio of PC(32∶1)+K to PC(32∶0) (Figure 3n) and the ratio of PC(34∶1)+K to PC(34∶0)+K (Figure 3o) did not show a statistical difference between these areas (p = 1.24E-02 and p = 0.690, respectively). Therefore, 4 out of 6 molecular species of MUFA-PC and SFA-PC showed higher intensities in the cancer cell areas, while none of the species showed lower intensity. Therefore, MUFA-PC with acyl chains of 36 carbons exhibited significantly high intensity compared to the SFA-PCs, and MUFA-PC with 32 acyl carbons showed the similar tendency although it was not accompanied by statistical significance.

In order to confirm how the lipids tended to be distributed by considering only the sections that had both cancer regions and reference regions, the ratios of MUFA-PCs to SFA-PCs were also compared between the cancerous areas and the reference areas by considering paring of the two areas on the same tissue sections. The number of samples that we could set 3 ROIs in the cancerous areas and 3 ROIs in the reference areas for each section was 6. The ratios of MUFA-PCs to SFA-PCs were calculated in the same way and analyzed by a paired *t*-test. Our results showed the same tendency as for the analysis using all of the sections; the ratios of PC(36∶1)+K/PC(36∶0)+K was significantly higher in the cancerous areas (p = 3.66E-03; Figure S2c).

### High Expression of SCD1 Protein in Cancerous Areas

**Figure 4:**
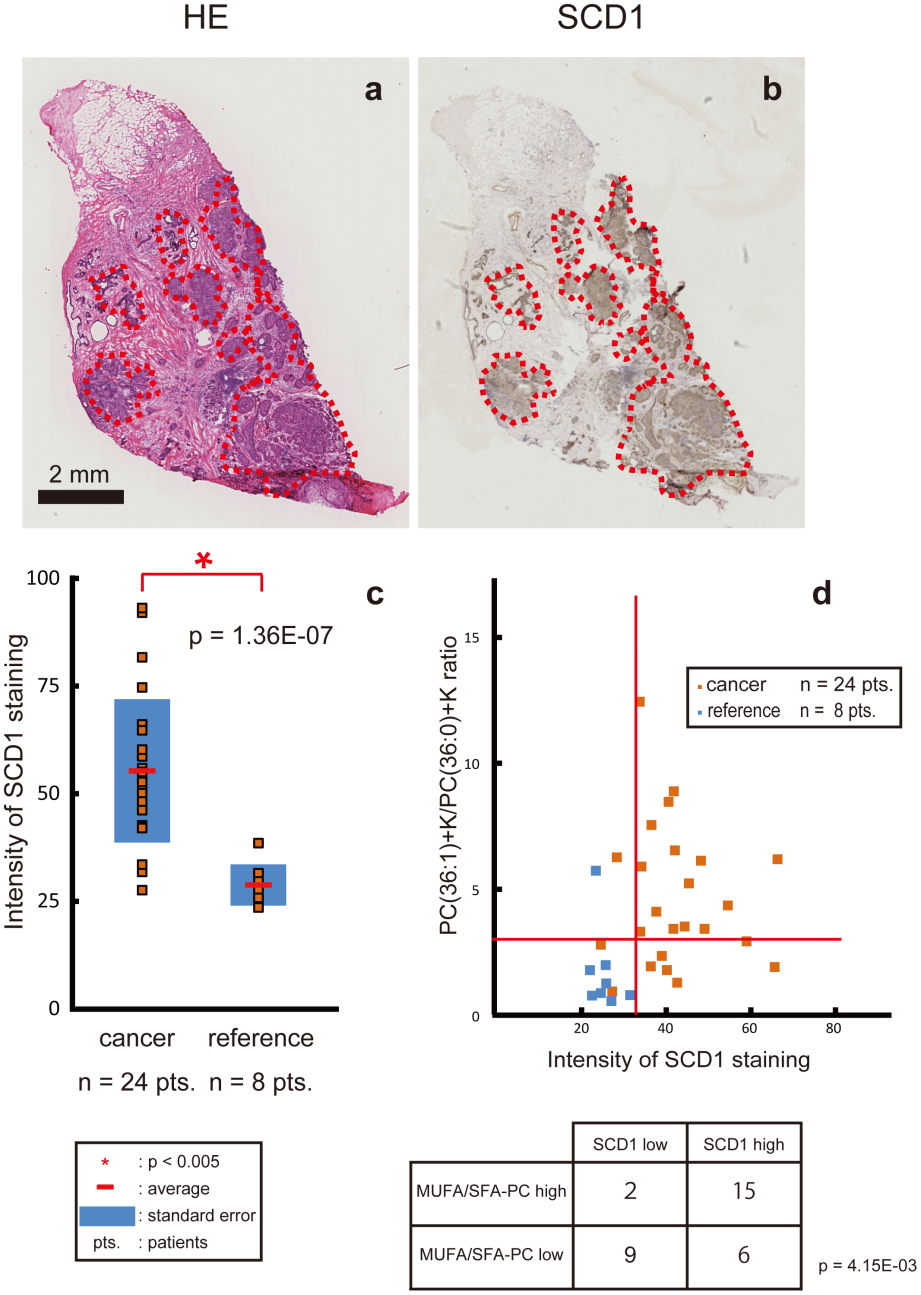
SCD1 protein was highly expressed in breast cancer tissues. (a) The areas that are circled with broken red lines in the hematoxylin and eosin stained image show cancerous areas. (b) Immunohistochemical staining for stearoyl-CoA desaturase-1 (SCD1) protein. (c) A plot of the intensities of SCD1 between the cancerous areas and the reference areas. (d) Values were plotted as SCD1 intensity on the x-axis and the MUFA-PCs/SFA-PCs ratio on the y-axis. The table shows the frequency of the subjects involved in each quadrant.

Past studies using cancer cell lines reported an important correlation between cancer cell proliferation and SCD1 [17], [28], [29]. We therefore examined whether the maldistribution of MUFA-PCs in the cancerous areas corresponded to SCD1 protein expression. Immunohistochemical staining revealed that SCD1 protein accumulated to a great degree in the cancerous areas (Figure 4a to c; p = 1.36E-07). We also examined whether the high ratio of MUFA-PC to SFA-PC coincided with high SCD1 expression by analyzing the frequencies of the quadrants in which the values of the subjects were plotted (Figure 4d, and Figure S3a and Sb). A biased distribution was confirmed by the chi-squared test in the case of PCs with 36 acyl carbons when a borderline was set so that the SCD1 intensity = 38 and the ratio = 3.5 (Figure 4d; p = 4.15E-03).

### The Ratios of MUFA-PCs Compared to LPCs were Significantly Higher for the Cancerous Areas

**Figure 5:**
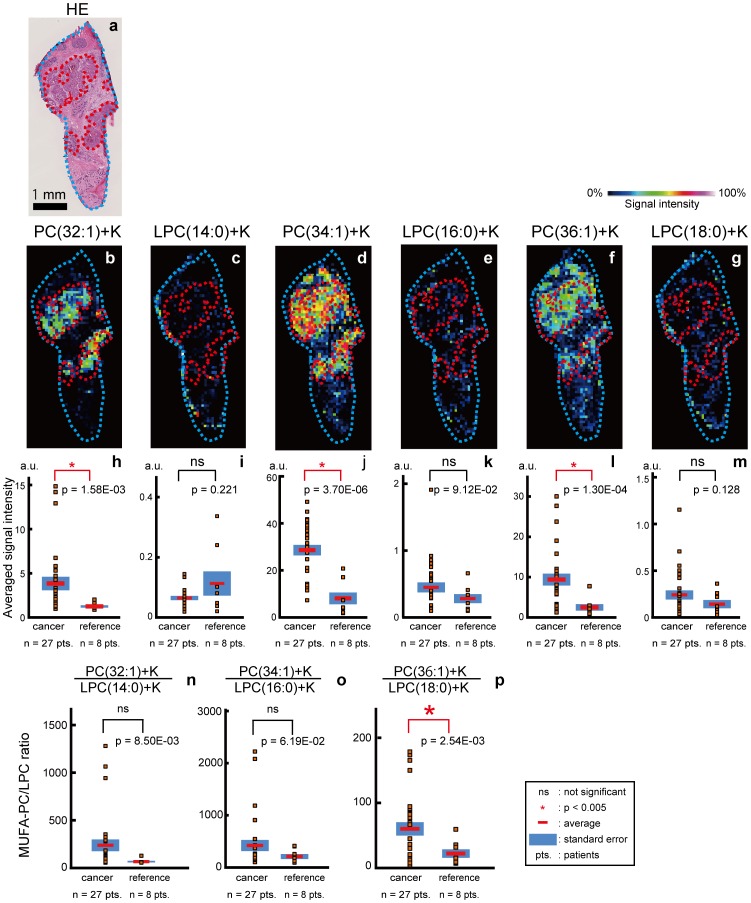
The ratios of MUFA-PCs to LPCs were significantly higher in cancerous areas than in reference areas. (a) The areas that are circled with red show the cancerous areas in the hematoxylin and eosin stained image. (b) A distribution image of PC(32∶1)+K. (c) A distribution image of LPC(14∶0)+K. (d) A distribution image of PC(34∶1)+K. (e) A distribution image of LPC(16∶0)+K. (f) A distribution image of PC(36∶1)+K. (g) A distribution image of LPC(18∶0)+K. (h) Plot of the intensities of PC(32∶1)+K. (i) Plot of the intensities of LPC(14∶0)+K. (j) Plot of the intensities of PC(34∶1)+K. (k) Plot of the intensities of LPC(16∶0)+K. (l) Plot of the intensities of PC(36∶1)+K. (m) Plot of the intensities of LPC(18∶0)+K in 35 ROIs. (n) Plot of the ratios of PC(32∶1)+K to LPC(14∶0)+K. (o) Plot of the ratios of PC(34∶1)+K to LPC(16∶0)+K. (p) Plot of the ratios of PC(36∶1)+K to LPC(18∶0)+K.

The production of MUFA-PCs is also catalyzed by lysophosphatidylcholine acyltransferases (LPCATs), which transfer MUFAs to LPCs [30] (Figure 1). The amount of LPCs is considerably balanced through its use by LPCAT and its supply via the metabolization of PC by phospholypases A1 and A2 (plA1 and plA2) as well as phospholipid:diacylglycerol acyltransferases [31], [32]. We next examined if this pool of LPCs showed a characteristic distribution in the cancer tissues in order to confirm that the accelerated synthesis was not due to the abundance of LPCs acting as substrates (Figure 5). A distribution of LPC(14∶0)+K, LPC(16∶0)+K and LPC(18∶0)+K bias toward the cancerous areas was not observed (Figures 5c, e, g, i, k, and m; p = 0.221, p = 9.12E-02, and p = 0.128, respectively). To estimate the balance of PC synthesis and metabolization, we compared the ratios of MUFA-PC to LPC in the cancerous areas and the reference areas. The ratio of PC(36∶1)+K to LPC(18∶0)+K (Figure 5p) was significantly higher in the cancerous areas than in the reference areas (p = 2.54E-03) and the ratios of PC(32∶1)+K to LPC(14∶0)+K and PC(34∶1)+K to LPC(16∶0)+K in the cancerous areas showed a tendency to be higher than those in the reference areas (Figure 5n and o; p = 8.50E-03 and p = 6.19E-02, respectively). Bias toward the synthesis of MUFA-PCs in the remodeling/degrading pathway in the cancerous areas was indicated.

The ratios of MUFA-PCs to LPCs in the cancerous area and the reference area located in the identical sections were also analyzed using the 6 tissue sections. The ratios of MUFA-PCs to LPCs were calculated and analyzed by using a paired *t*-test. Here as well, we obtained results that showed the same tendency as seen for the analysis using all of the sections: all of the ratios of MUFA-PCs to LPCs were significantly higher in the cancerous areas (Figures S4a to c; p = 1.37E-05 and p = 3.63E-03, and p = 9.79E-03, respectively).

### The Ratios of MUFA-PCs Compared to SFA-PCs and to LPCs in the Cancerous Areas Observed through the Differences in Expressions of ER, HER2, and Ki67

**Figure 6:**
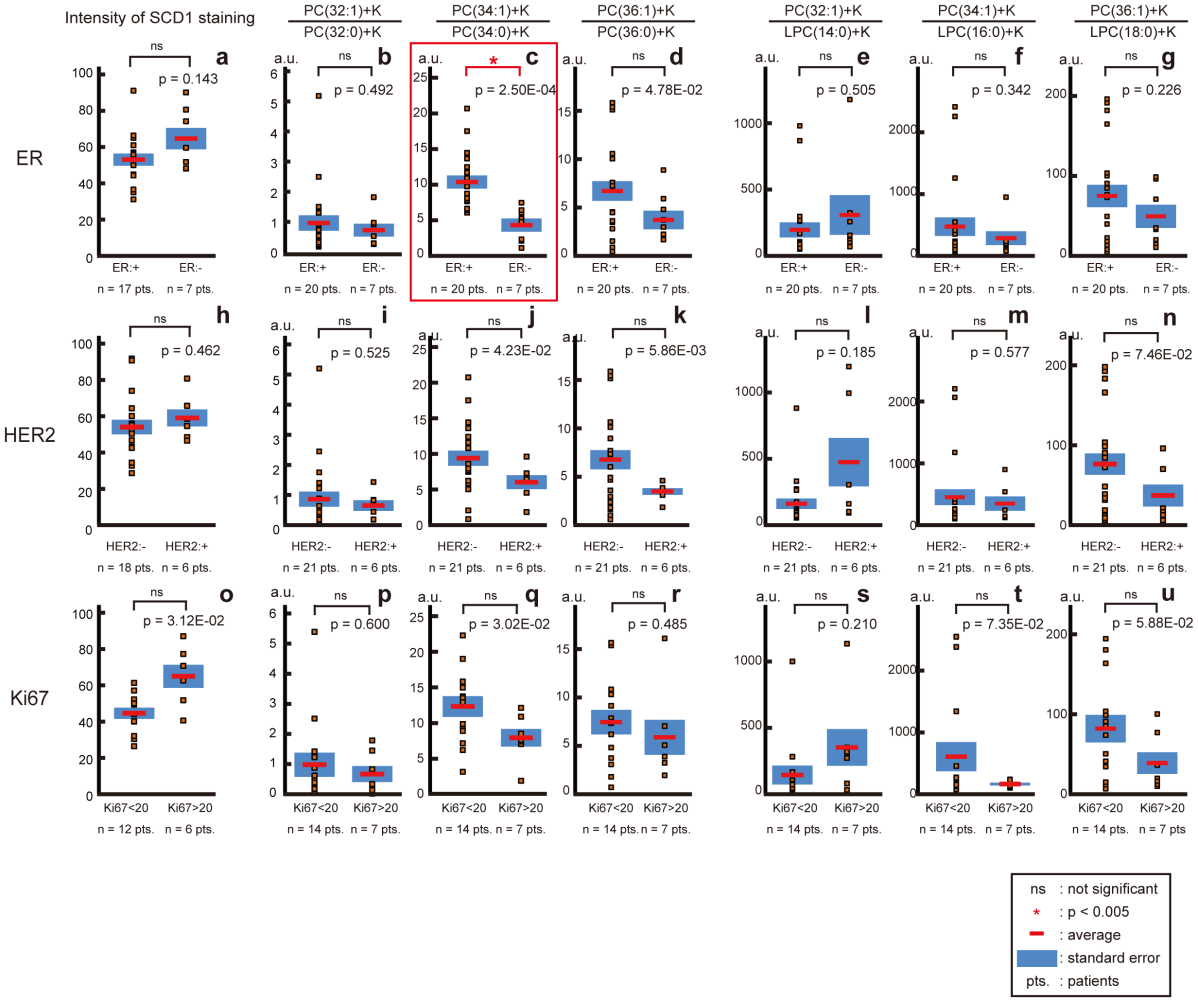
SCD1 expression and the ratios of MUFA-PCs to SFA-PCs and LPCs for differences in ER, HER2, and Ki67 expression. The intensities of SCD staining (a, h, o) and the ratios of MUFA-PC compared to SFA-PC (b–d, i–k, p–r) and LPC (e–g, l–n, s–u) were shown by the differences in ER (a–g), HER2 (h–n), and Ki67 (o–u). All of the cancer lesions were divided into two groups according to the differences in ER, HER2, and Ki67 expression and lesions with less aggressiveness are shown on the left side of each graph.

Prognosis and survival rates for breast cancer vary greatly depending on the cancer subtype. ER negativity and HER2 positivity are known to be poor prognostic markers [33]. Ki67 is a nuclear protein that is present during all active phases of the cell cycle and is known to be a proliferation marker of many malignancies including breast cancer [34]. Therefore, we examined the SCD1 expression and the ratios of MUFA-PC compared to SFA and LPC considering the differences in expression of ER, HER2, and Ki67 (Figure 6). All of the cancerous lesions were divided into two groups: a less aggressive group that was ER positive, a group that was HER2 negative or showed low Ki67 expression, and a group that exhibited more aggressive ER negative, HER2 positive, or high Ki67 expression. Among the comparisons between the pairs of groups, only the comparison of the ratio of MUFA-PC [PC(34∶1)] to SFA-PC [PC(34∶0)] between the ER positive and negative specimens showed statistical significance: the ER positive lesions were higher than the ER negative lesions (Figure 6c; p = 2.50E-04). The other pairs including those for comparison in terms of SCD1 intensity did not show statistical differences while the lesions with negative HER2 showed a higher tendency ratio than did the HER2 positive lesions (Figure 6k; p = 5.86E-03).

## Discussion

The visualization of molecules has contributed greatly to the characterization of clinical specimens derived from breast cancer patients. For example, the examination of protein expression of receptors such as HER2 and ER has been frequently used in classification [35], [36], and fluorescent in situ hybridization is used to detect the amplification of oncogenes such as myc and HER2 [37]. On the other hand, lipids in clinical specimens have been investigated by nonspecific histochemistry using chemicals such as oil red [38]. This study firstly utilized MALDI-IMS to analyze clinical breast cancer specimens to visualize lipid discriminating acyl chain structures, quantitatively analyzed the region-specific signal intensities of multiple lipid molecules, and proposes a new methodology to clinically research breast cancer.

The system of PC synthesis that we focused on in this report was a remodeling pathway, which is one of two routes where PCs are synthesized: the de novo pathway (known as the Kennedy pathway) and the remodeling pathway (Land’s pathway) [39]. In the remodeling pathway, MUFAs that are endogenously produced from SFAs through the influence of SCD1 are converted into MUFA-PCs. Reports have shown varying levels of SCD1 expression in different tumor samples, including breast cancer [19], [27], and higher SCD1 expression in breast cancer has been recently proposed as a poor prognostic marker [18]. To the best of our knowledge, this study is the first report showing that SCD1 is expressed in high levels in breast cancer cells in conjunction with a relatively high amount of MUFA-PCs. Among the 3 ratios of MUFA-PCs to SFA-PCs, only the ratio of PC(36∶1) to PC(36∶0) were found to be significantly related to the high SCD1 expression; this observation can be attributed to the SCD1 preferentially desaturating FA(18∶0) over FA(16∶0) [40]. The result that only the ratios of PC(36∶1) to PC(36∶0) in cancerous areas were higher might be explained by higher affinity of SCD1 to FA(18∶0).

On the other hand, the result of a sub-analysis of the ratio of MUFA-PC [PC(34∶1)] to SFA-PC [PC(34∶0)] with respect to ER expression was not consistent with the SCD1 expression pattern: SCD1 expression did not show a significant difference between the ER positive groups and ER negative groups. This result could be explained by 2 possible reasons. First, there may be other factors that regulate the relative amount of MUFA-PCs. The LPCATs that catalyze the insertion of MUFAs into LPCs might be one of the candidates. [39], [41]. We found from a microarray analysis that LPCAT3 is expressed at higher levels in ER positive tissues than in ER negative tissues [42], [43], which is consistent with the correlation between ER status and the MUFA-PC/SFA-PC ratio that was observed in the present study. LPCAT4 is one LPCAT isoform that shows a higher specificity to FA(18∶1) [44]. LPCAT4 may be acting as a regulating factor in this study as well, although no comparative studies on LPCAT4 expression between ER-positive and ER-negative breast cancer lesions have been reported. Second, a greater activity of the SCD1 enzyme in ER-positive cells than that in ER-negative cells could cause an excessive production of MUFA-PCs in these cells. As the endogenous mechanisms to regulate the enzymatic activity of SCD1 are poorly understood, further studies on the SCD1 activity and its regulatory mechanism might be warranted.

We alternatively defined tumor aggressiveness on the basis of immunohistochemical staining of pathological markers, ER negativity, HER2 positivity, and high Ki67 expression. Because exogenous HER2 overexpression induces upregulation of fatty acid synthase (FASN) in breast cancer cells, the pathway involving the HER2 receptor is generally considered to regulate lipogenic enzymes [45]. The activation of lipid metabolism in tumor cell proliferation is widely accepted [2], [3]; thus, the correlation of Ki67 expression level and PC compositions were expected. However, the ratios presented in Figure 6 did not show significant differences between high- and low-expression groups of HER2 and Ki67. We therefore could not prove the effect of the expression of these molecules on the acyl chains in PCs. Regarding the result that only the ratio of PC(34∶1)/PC(34∶0) showed significant differences between ER-positive and ER-negative groups, we propose the following mechanism. SCD1 predominantly converts FA(18∶0) to FA(18∶1) [40] and LPCATs combine FA(18∶1) and LPC(16∶0) [44], putatively the most abundant LPC, [46], [47] to generate PC(34∶1). Therefore, PC(34∶1) might be preferentially affected by the alteration of expression and activity of the enzymes. However, further studies on this mechanism are warranted to confirm these statements that are based on propositions.

In this study, we analyzed the PCs of specific carbon chain lengths and saturation to characterize the specimens. Since polyunsaturated fatty acids have been argued to function in cancer hallmarks by maintaining and disrupting membrane microstructures and by tuning signal transductions [48], [49], further studies involving PCs that are composed of fatty acids with longer chains and greater degrees of unsaturation might lead to a better understanding of the function of phospholipids in cancer pathogenesis.

Currently, MALDI-IMS as a technique is developing and is expected to have enough resolution to allow investigators to define and analyze smaller areas. If MALDI-IMS could be improved, it would be a useful tool for exploring the mechanisms of carcinogenesis and cancer metastasis. The analysis of cancer stem cells like treatment-resistant breast cancer cells that share small populations of thousands of cells in lesions [50] might be possible. A combination of improved MALDI-IMS and immunohistochemical staining might reveal a correlation between lipid composition and receptor expression, which has been scarcely reported [51]. MALDI-IMS has the potential to be a favorable tool to study breast cancer tissues with molecular heterogeneity [52]. We are attempting to improve the capabilities of MALDI-IMS by adopting new agents as a matrix [53], by using mass microscopes [54] and so on to perform these analyses in the near future.

### Conclusions

IMS was used to successfully visualize molecular species of PCs and LPCs in human breast cancer tissue specimens. Some MUFA-PCs and SFA-PCs [PC(32∶1), PC(34∶1), PC(36∶1), and PC(34∶0)] were relatively localized in cancerous areas rather than the rest of the sections, while LPCs were equally distributed. Some ratios of MUFA-PCs to SFA-PCs or LPCs [PC(36∶1)/PC(36∶0) and PC(36∶1)/LPC(18∶0)] were higher in the cancerous areas than the references. The high expression of SCD1 in the cancerous areas was indicative that this enzyme partially mediates the production of MUFA-PCs that were observed in these areas. The analysis of the relative amount of MUFA-PC [PC(34∶1) compared to PC(34∶0)] through the differences in ER expression suggested the importance of other factors that regulate lipid composition.

## Supporting Information

Figure S1 **Samples after IMS analysis were severely damaged and closely resembled the adjacent tissue sections microscopically.** (a) Adjacent tissue sections that were used for counter staining with hematoxylin and eosin (HE). (b) HE stained samples after IMS analysis.
(TIF)

Figure S2 **Comparison of MUFA-PCs to SFA-PCs ratios between cancerous and reference areas on same tissue sections.** (a) Plot of the ratios of PC(32∶1)+K to PC(32∶0)+K. (b) Plot of the ratios of PC(34∶1)+K to PC(34∶0)+K. (c) Plot of the ratios of PC(36∶1)+K to PC(36∶0)+K.
(TIFF)

Figure S3 **Plot of SCD1 intensity and the MUFA-PCs/SFA-PCs ratio.** The values from the subjects were plotted as SCD1 intensity on the x-axis and the MUFA-PCs/SFA-PCs ratio on the y-axis (a, b). A table shows the frequency of the subjects involved in each quadrant divided by the borderlines (a; p = 9.68E-03). The threshold with which bias in the frequencies in the quadrants were proven was not discovered for these molecules. The threshold and the frequency on PCs with 32 acyl carbons were presented since the examination showed relatively low p value.
(TIFF)

Figure S4 **Comparison MUFA-PCs to LPCs ratios between cancerous and reference areas on same tissue sections.** (a) Plot of the ratios of PC(32∶1)+K to LPC(14∶0)+K. (b) Plot of the ratios of PC(34∶1)+K to LPC(16∶0)+K. (c) Plot of the ratios of PC(36∶1)+K to LPC(18∶0)+K.
(TIFF)
