# Correction: Human Breast Cancer Tissues Contain Abundant Phosphatidylcholine(36∶1) with High Stearoyl-CoA Desaturase-1 Expression

**DOI:** 10.1371/annotation/63c5359c-b1d2-44d8-944f-7bde0d6ec946

**Published:** 2013-09-09

**Authors:** Yoshimi Ide, Michihiko Waki, Takahiro Hayasaka, Tomohisa Nishio, Yoshifumi Morita, Hiroki Tanaka, Takeshi Sasaki, Kei Koizumi, Ryoichi Matsunuma, Yuko Hosokawa, Hiroyuki Ogura, Norihiko Shiiya, Mitsutoshi Setou

There was an error in the second to last sentence in the Abstract. The correct sentence is: The ratios of the signal intensity of PC(34:1) to that of PC(34:0) was higher in the lesions with positive ER expression. 

