# Supplementary figures and images for: Human Breast Cancer Tissues Contain Abundant Phosphatidylcholine(36∶1) with High Stearoyl-CoA Desaturase-1 Expression
Source: PLoS One. 2013 Apr 16;8(4):e61204. doi: 10.1371/journal.pone.0061204 (PMC3629004; doi:10.1371/journal.pone.0061204)

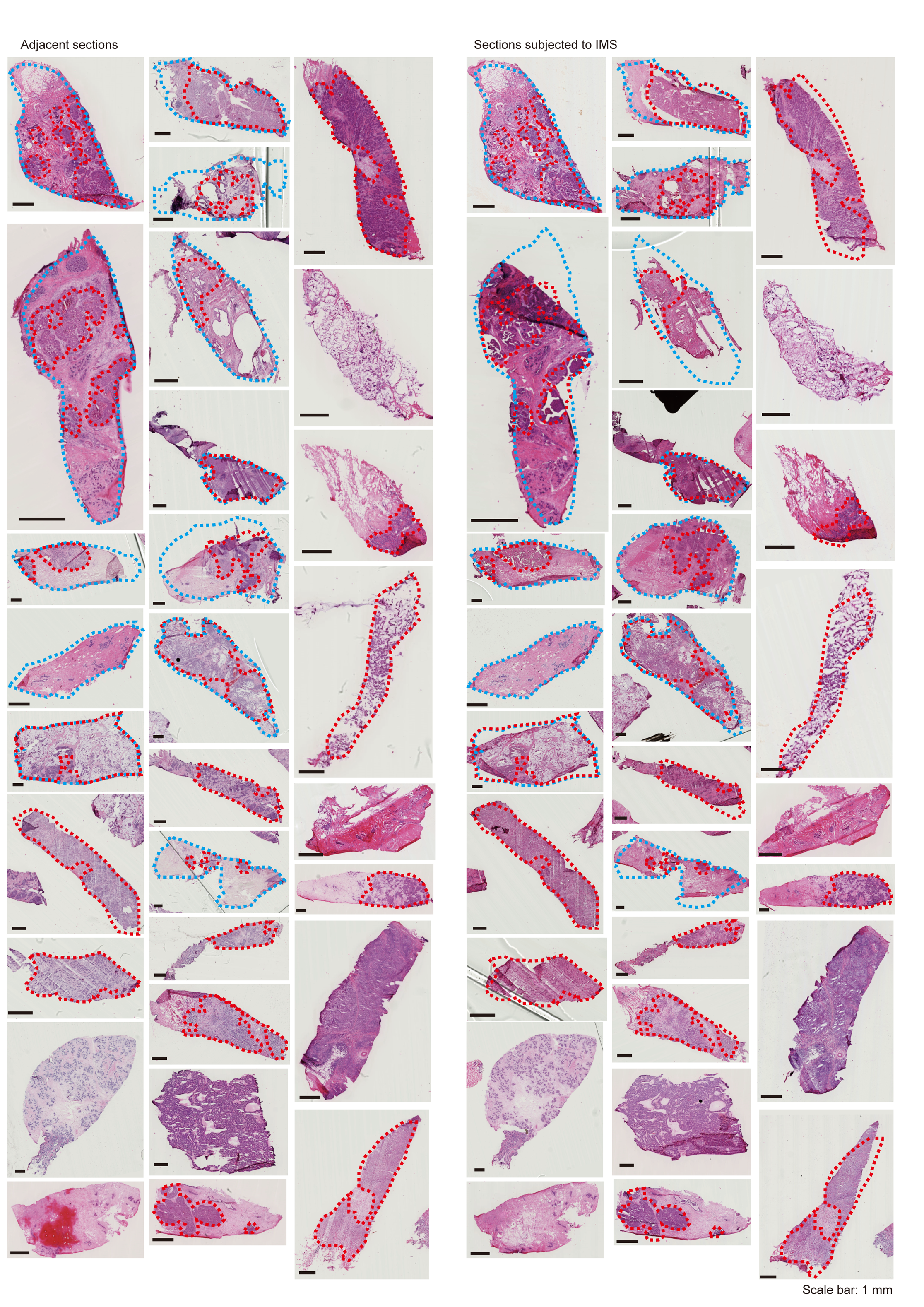

Supplement: Figure S1 — Samples after IMS analysis were severely damaged and closely resembled the adjacent tissue sections microscopically. (a) Adjacent tissue sections that were used for counter staining with hematoxylin and eosin (HE). (b) HE stained samples after IMS analysis. (TIF) [file pone.0061204.s001.tif]

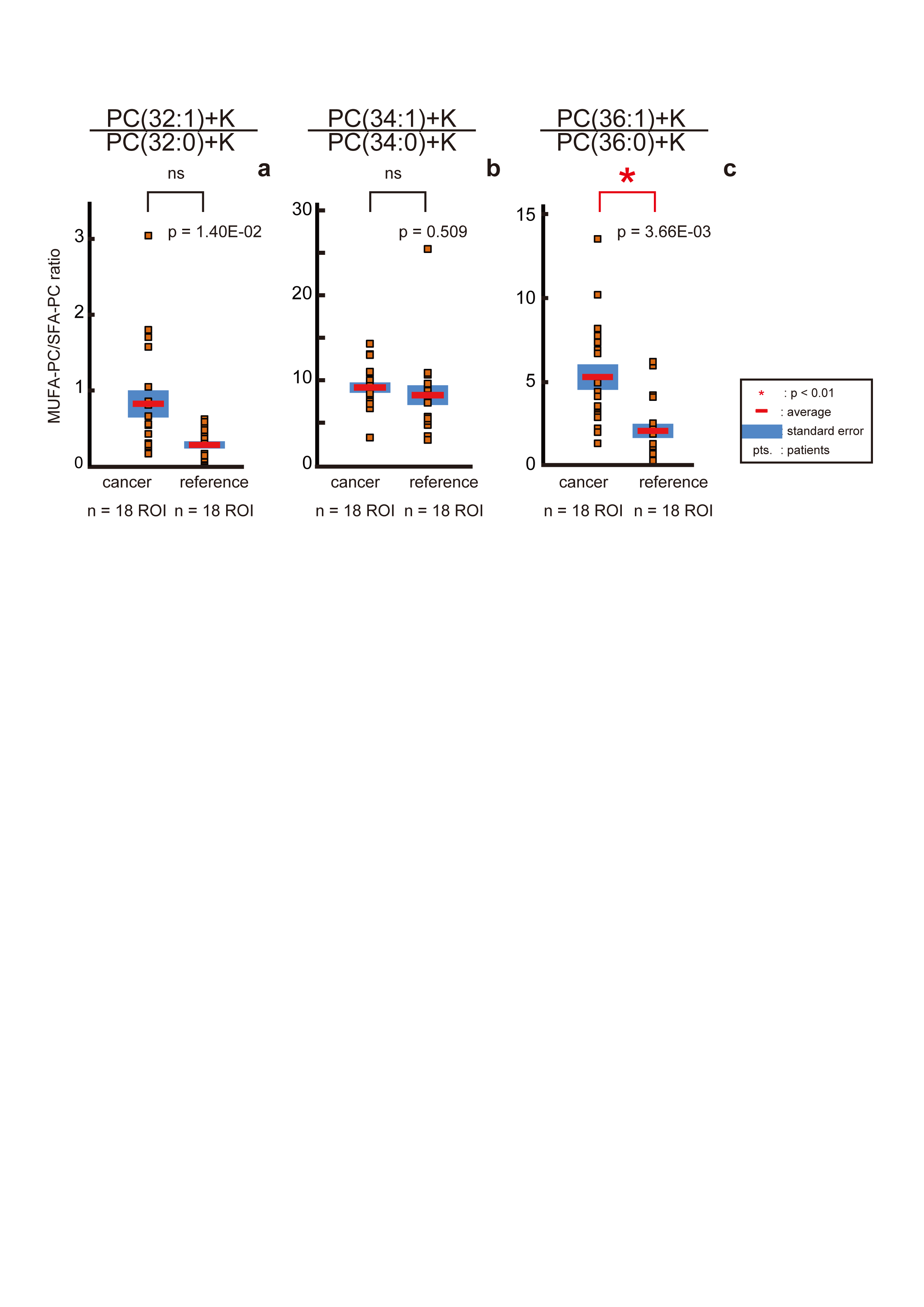

Supplement: Figure S2 — Comparison of MUFA-PCs to SFA-PCs ratios between cancerous and reference areas on same tissue sections. (a) Plot of the ratios of PC(32∶1)+K to PC(32∶0)+K. (b) Plot of the ratios of PC(34∶1)+K to PC(34∶0)+K. (c) Plot of the ratios of PC(36∶1)+K to PC(36∶0)+K. (TIFF) [file pone.0061204.s002.tiff]

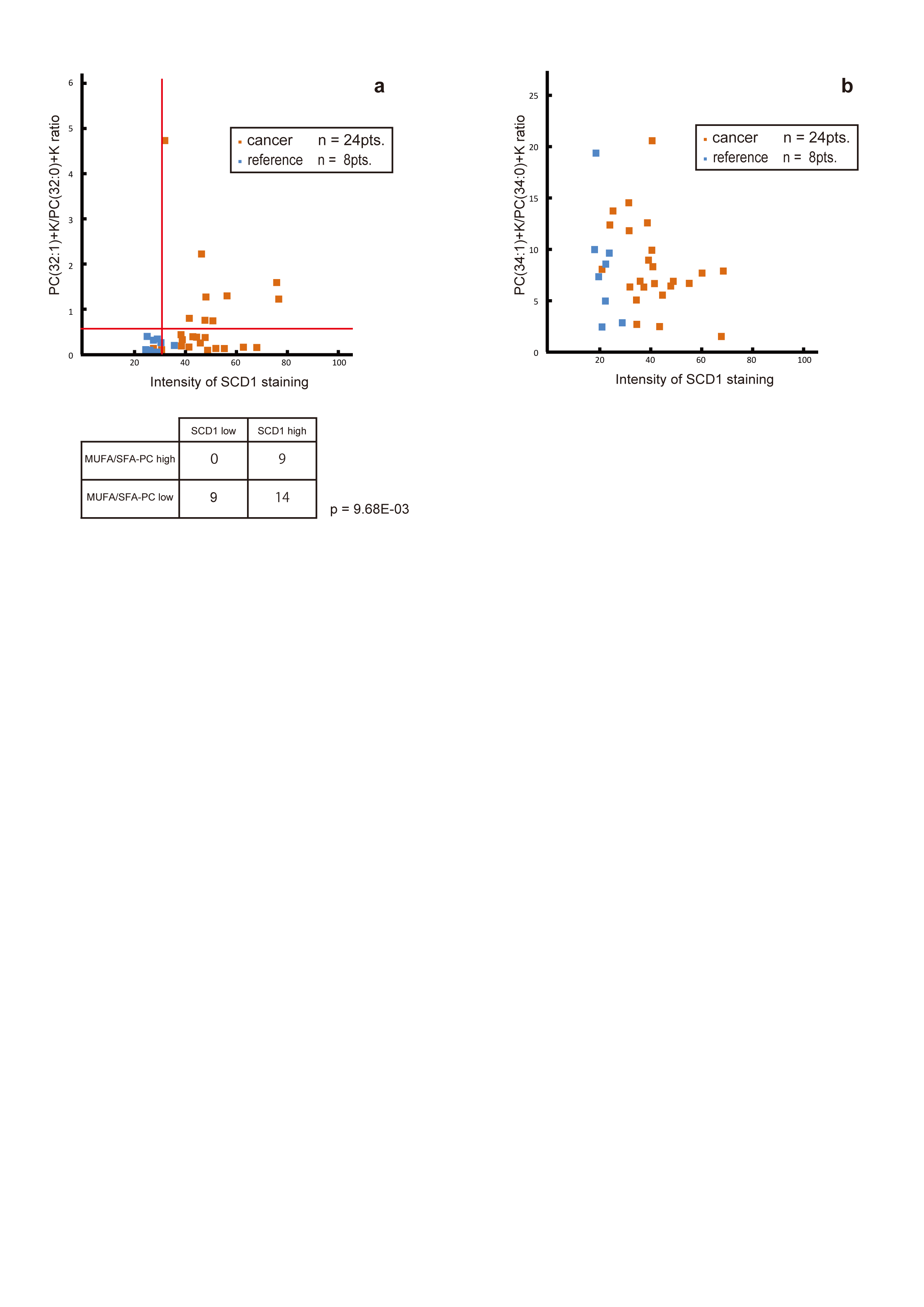

Supplement: Figure S3 — Plot of SCD1 intensity and the MUFA-PCs/SFA-PCs ratio. The values from the subjects were plotted as SCD1 intensity on the x-axis and the MUFA-PCs/SFA-PCs ratio on the y-axis (a, b). A table shows the frequency of the subjects involved in each quadrant divided by the borderlines (a; p = 9.68E-03). The threshold with which bias in the frequencies in the quadrants were proven was not discovered for these molecules. The threshold and the frequency on PCs with 32 acyl carbons were presented since the examination showed relatively low p value. (TIFF) [file pone.0061204.s003.tiff]

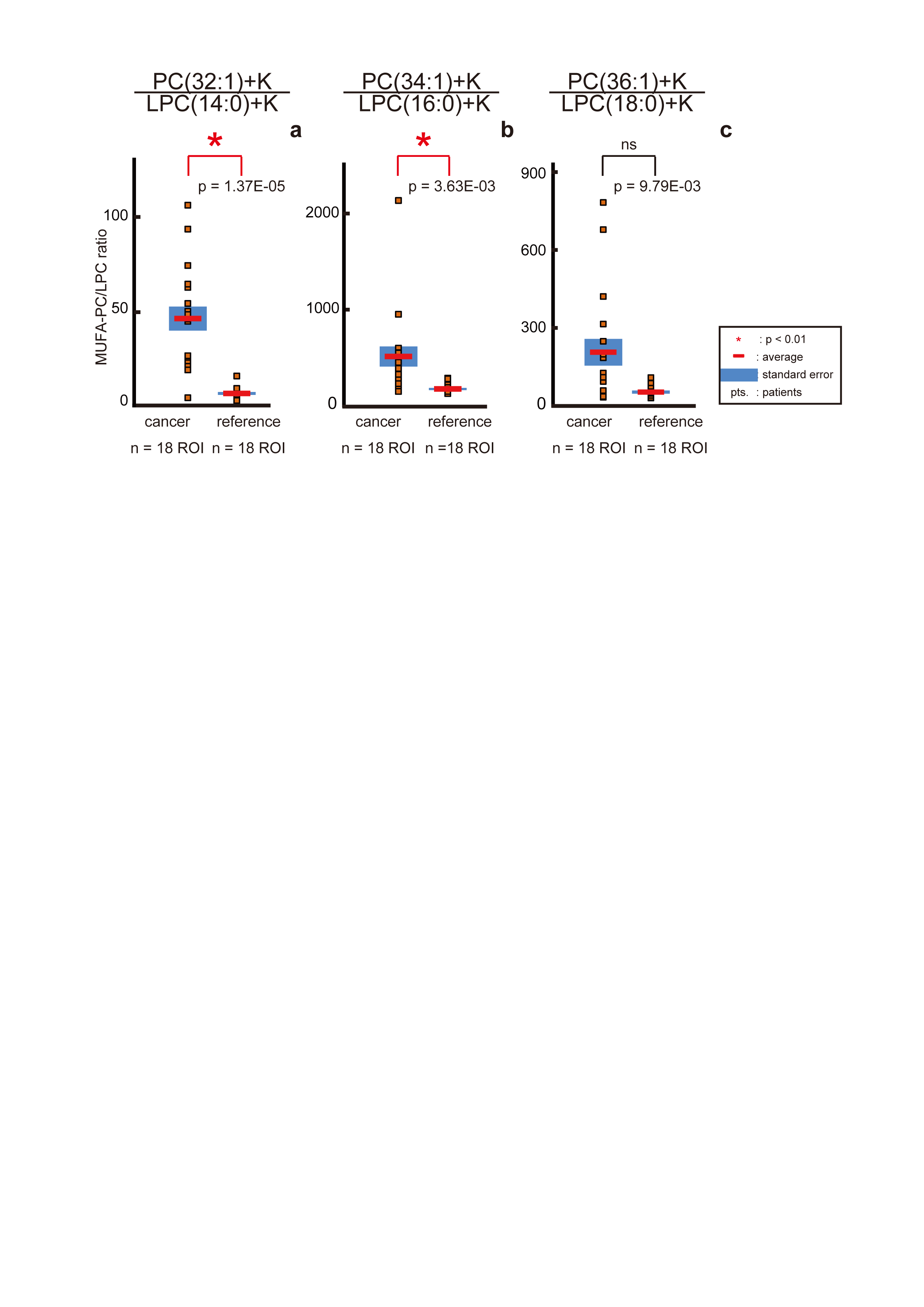

Supplement: Figure S4 — Comparison MUFA-PCs to LPCs ratios between cancerous and reference areas on same tissue sections. (a) Plot of the ratios of PC(32∶1)+K to LPC(14∶0)+K. (b) Plot of the ratios of PC(34∶1)+K to LPC(16∶0)+K. (c) Plot of the ratios of PC(36∶1)+K to LPC(18∶0)+K. (TIFF) [file pone.0061204.s004.tiff]
